# DsbA-L Ameliorates Renal Injury Through the AMPK/NLRP3 Inflammasome Signaling Pathway in Diabetic Nephropathy

**DOI:** 10.3389/fphys.2021.659751

**Published:** 2021-04-30

**Authors:** Ming Yang, Shilu Luo, Na Jiang, Xi Wang, Yachun Han, Hao Zhao, Xiaofen Xiong, Yan Liu, Chanyue Zhao, Xuejing Zhu, Lin Sun

**Affiliations:** ^1^Department of Nephrology, Hunan Key Laboratory of Kidney Disease and Blood Purification, The Second Xiangya Hospital, Central South University, Changsha, China; ^2^Department of Nutrition, Xiangya Hospital, Central South University, Changsha, China

**Keywords:** NLRP3 inflammasome, DsbA-L, AMPK, diabetic nephropathy, inflammation

## Abstract

NLRP3-mediated inflammation is closely related to the pathological progression of diabetic nephropathy (DN). DsbA-L, an antioxidant enzyme, plays a protective role in a variety of diseases by inhibiting ER stress and regulating metabolism. However, the relationship of DsbA-L with inflammation, especially the NLRP3 inflammasome, has not been examined. In this study, we note that activation of the NLRP3 inflammasome and exacerbated fibrosis were observed in the kidneys of diabetic DsbA-L-knockout mice and were accompanied by decreased phosphorylation of AMP-activated protein kinase (AMPK). Moreover, correlation analysis shows that the phosphorylation of AMPK was negatively correlated with NLRP3 expression and tubular damage. In addition, the decreased AMPK phosphorylation and NLRP3 activation induced by high glucose (HG) in HK-2 cells could be alleviated by the overexpression of DsbA-L. Interestingly, the protective effect of DsbA-L was eliminated after treatment with compound C, a well-known AMPK inhibitor. Our findings suggest that DsbA-L inhibits NLRP3 inflammasome activation by promoting the phosphorylation of AMPK.

## Introduction

Type 2 diabetes mellitus (DM2) is a metabolic disease characterized by increased blood glucose levels and insulin resistance. With the improvement of people’s standards of living, the incidence of diabetes is increasing, and it was estimated that 415 million people worldwide were living with DM2 in 2015 (Saeedi et al., 2019; O’Brien et al., 2020). Long-term uncontrolled hyperglycemia leads to a series of microvascular complications, such as diabetic retinopathy (DR) (Limwattanayingyong et al., 2020) and diabetic nephropathy (DN) (Wei et al., 2021). In developed countries, DN has become the leading cause of end-stage renal disease (ESRD), which causes a heavy medical burden on society (Alicic et al., 2017; Xue et al., 2017; Umanath and Lewis, 2018). Many factors have been proven to be involved in the development of DN, such as abnormal mitophagy (Yang et al., 2020) and oxidative stress (Yang et al., 2017). Moreover, there is considerable evidence that the abnormal induction of NLRP3-mediated inflammation plays a role in the development of DN that cannot be ignored (Wada and Makino, 2016; Fu et al., 2017).

As the most widely studied inflammasome, the nucleotide binding and oligomerization domain-like receptor family pyrin domain-containing 3 (NLRP3) inflammasome is shown to be involved in a variety of diseases, including DN (Hutton et al., 2016; Ding et al., 2018). A large number of infiltrating macrophages and activation of the NLRP3 inflammasome are observed in renal biopsy tissues from diabetic patients (Chen et al., 2013; Zheng and Zheng, 2016). Interestingly, the level of NLRP3 mRNA is also notably positively correlated with the urinary albumin/creatinine ratio, serum creatinine (El-Horany et al., 2017). Similarly, activation of the NLRP3 inflammasome is also observed in mice with STZ-induced DN (Hou et al., 2020) or in db/db mice (Yi et al., 2017), and inhibition of NLRP3 inflammasome activation by chemical treatment or gene knockout alleviated renal inflammation and pathological progression in mice with DN (Ding et al., 2018; Wu et al., 2018). These results suggest that NLRP3 inflammasome activation plays an indispensable role in the pathogenesis of DN. Abnormalities in mitochondrial function and the NF-κB pathway are shown to be involved in the activation of the NLRP3 inflammasome in DN (Samra et al., 2016; Han et al., 2018). However, these pathways do not adequately explain the activation of the NLRP3 inflammasome in the kidney of DN.

AMP-activated protein kinase (AMPK) is the primary sensor of the cellular energy balance and the basic regulator of cellular carbohydrate and fat metabolism and ATP maintenance and synthesis (Ma et al., 2019). Recent studies show that AMPK plays a key role in the activation of the NLRP3 inflammasome. Yang F. et al. (2019) demonstrate that metformin could alleviate NLRP3-mediated inflammation in the diabetic myocardium by promoting AMPK phosphorylation. Moreover, cordycepin (CRD), extracted from cordyceps militaris, could alleviate NLRP3-mediated pancreatic inflammation by promoting AMPK activation (Ma et al., 2019).

DsbA-L, a 25-kDa protein, was initially found in the matrix of rat liver mitochondria (Harris et al., 1991). DsbA-L can also inhibit ER stress and improve adiponectin secretion by interacting with the ER chaperone Ero1-L (Liu et al., 2015). Moreover, in a previous study, we demonstrated that DsbA-L plays a renal protective role by alleviating lipid deposition by activating AMPK in the kidneys of patients with DN (Chen et al., 2019). However, the relationship between DsbA-L and NLRP3-mediated inflammation is unclear.

In the present study, we find that activation of the NLRP3 inflammasome and exacerbation of renal fibrosis are observed in the kidneys of diabetic DsbA-L knockout mice and are accompanied by decreased AMPK phosphorylation. In addition, we demonstrate that DsbA-L exerts an anti-inflammatory effect by promoting the phosphorylation of AMPK.

## Materials and Methods

### Animal Model

DsbA-L^+/–^ mice (C57BL/6 background) were generated by Shanghai Bioray Laboratory Inc., China (Gene ID, 76263) (Chen et al., 2019). DsbA-L^–/–^ mice were obtained by crossing a DsbA-L^+/–^ female mouse with a DsbA-L^+/–^ male mouse, and genotyping and Western blot analysis were used to determine DsbA-L gene disruption as described previously (Chen et al., 2019). The control group included the wild-type littermate. At 8 weeks of age, the mice were fed a high-fat diet (HFD) for 4 weeks to induce insulin resistance, followed by intraperitoneal injection of 50 mg/kg/day streptozotocin (STZ) for five consecutive days. Twenty-four hours after the injection, random glucose measurements of blood collected from the tail vein showing values > 200 mg/L indicated successful establishment of the model (Chen et al., 2019). Then, HFD feeding was continued until 26 weeks of age. The mice were divided into four groups: the WT group, DsbA-L^–/–^ group, WT+HFD+STZ group, and DsbA-L^–/–^+HFD+STZ group (*n* = 3). All the mice were housed in a quiet environment with a constant temperature (22–26°C). All the experiments were approved by the Medical Ethics Committee of Central South University.

### Renal Histology

The renal tissues from the mice were fixed in 10% neutral formalin for 24 h. After being treated with alcohol and xylene at different concentration gradients, the renal tissues were finally embedded in paraffin. Renal paraffin sections (3-μm-thick sections) were used for hematoxylin and eosin (HE) and Masson’s trichrome staining to assess pathological changes in the kidneys as described previously (Yang M. et al., 2019).

### Cell Culture and Treatment

A human proximal tubular cell line (HK-2) was purchased from ATCC (United States) and cultured in medium containing 5 or 30 nM D-glucose in an environment with 5% CO_2_ at 37°C. Cells overexpressing DsbA-L were generated by transfection with the pcDNA3.1-DsbA-L plasmid and Lipofectamine 2000 reagent according to the manufacturer’s instructions (Chen et al., 2019).

### Western Blot Analysis

Proteins were extracted from mouse kidney or HK-2 cells with radioimmunoprecipitation assay (RIPA) buffer containing protease inhibitors. The protein concentrations were quantified by a BCA Protein Assay Kit (Beyotime Biotechnology, China). Equal amounts of proteins were subjected by Western blot analysis. The expression of the target proteins was analyzed by SDS-PAGE (8%) and using antibodies against FN (Abcam, ab2413, 1:1000), COL-1 (Abcam, ab260043, 1:1000), NLRP3 (Abcam, ab214185, 1:1000), p-AMPK (CST, 1:1000), AMPK (CST, 1:1000), and GAPDH (Proteintech, 60004-1-Ig, 1:50,000).

### Statistical Analysis

SPSS 20.0 software was used to perform the statistical analyses. The values are presented as the means ± SD, and the differences among the groups were compared by one-way ANOVA. Pearson’s analysis was used to test the correlation between two numerical variables. Statistical significance means *P-*value less than 0.05.

## Results

### Pathological Damage and Fibrosis Were Exacerbated in the Kidneys of Diabetic DsbA-L-Deficient Mice

**FIGURE 1:**
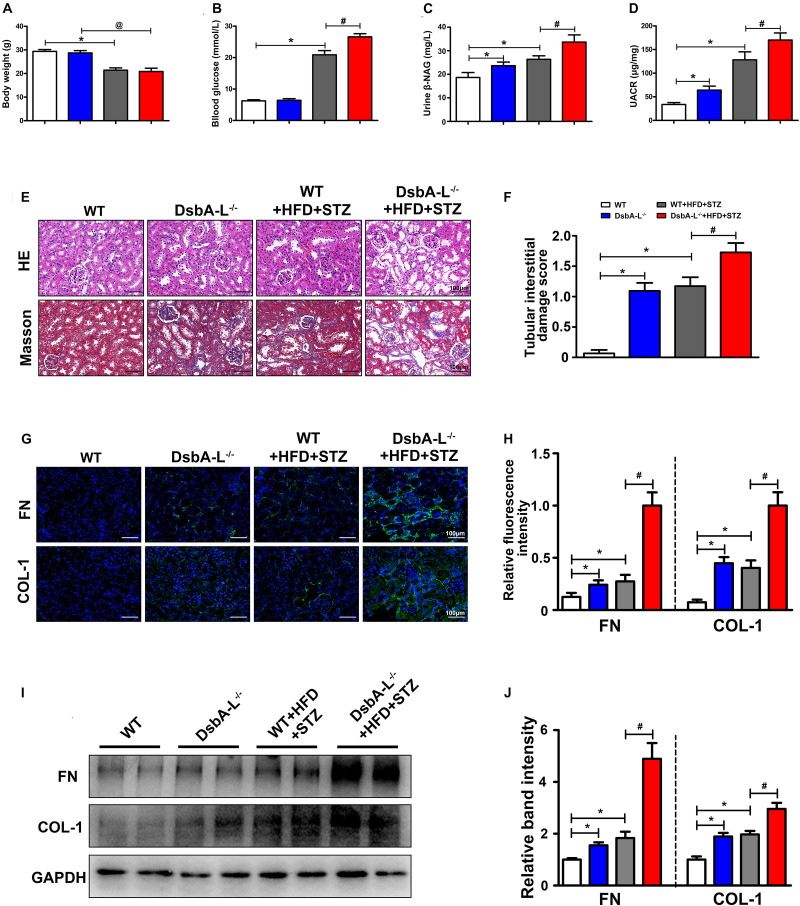
Pathophysiological changes and renal fibrosis in diabetic DsbA-L-deficient mice. Body weight **(A)**, blood glucose **(B)**, urine β-NAG **(C)**, and UACR **(D)** in each group. **(E)** HE and Masson staining were used to detect pathological changes in the kidneys. **(F)** The degree of tubular injury as assessed by the tubular interstitial damage score. **(G,H)** IHF staining with anti-fibronectin antibody (upper panel) and anti-collagen 1 antibody (lower panel) shows the renal fibrosis. **(I,J)** Western blot analysis reveals the expression of FN and COL-1. The values are the mean ± SD. *n* = 3/group. *p < 0.05 compared with the control group; #*p* < 0.05 compared with the diabetic group.

Western blot analysis revealed that DsbA-L expression was notably decreased in mice with DN compared with the control mice (Supplementary Figure 1A). A lower body weight was observed after STZ-induced diabetes was established (shown in Figure 1A). The blood glucose level was higher in the mice with DN although this increase was more dramatic in the DsbA-L^–/–^+HFD+STZ mice (shown in Figure 1B). What is more, the urine β-NAG and UACR levels were notably increased in the mice of DsbA-L^–/–^ and HFD+STZ-induced DN groups compared with those of the control group although they were further increased in the mice of the DsbA-L^–/–^+HFD+STZ group (shown in Figures 1C,D). HE and Masson staining revealed aggravating tubulointerstitial lesions (dilated lumen, bare nucleus, and interstitial fibrosis) in the kidneys of the DsbA-L^–/–^ or diabetic mice compared with those of the control mice although these pathological changes were further exacerbated in the kidneys of DsbA-L^–/–^+HFD+STZ mice (shown in Figure 1E). Visual quantification showed the highest score in the DsbA-L^–/–^+HFD+STZ group (shown in Figure 1F). The degree of renal fibrosis was detected by the fibrogenic proteins fibronectin (FN) and collagen type 1 (COL-1). Immunohistofluorescence (IHF) staining showed that the levels of FN and COL-1 expression (green area) in the tubulointerstitium were increased in the kidneys of DsbA-L^–/–^ or diabetic mice compared with those of control mice, and they were further increased in DsbA-L^–/–^+HFD+STZ mice (shown in Figures 1G,H). Similar results were also observed by Western blot analysis, and the DsbA-L^–/–^+HFD+STZ mice showed the most severe level of renal fibrosis (shown in Figures 1I,J).

### Increased Activation of NLRP3 Inflammasome in the Kidney of Diabetic DsbA-L-Deficient Mice

**FIGURE 2:**
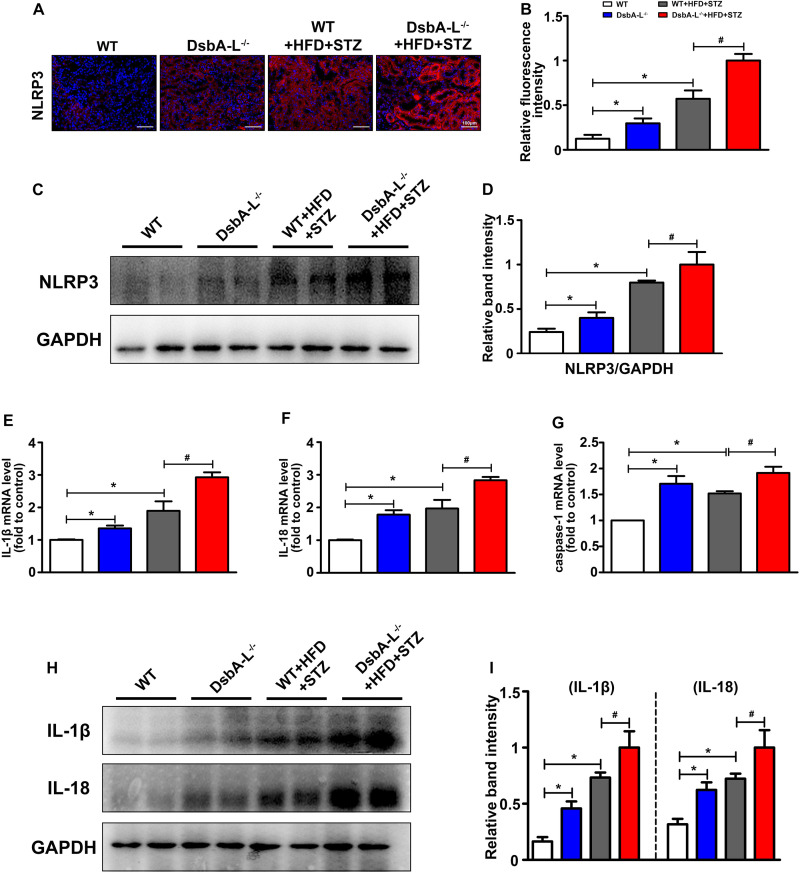
Activated NLRP3 inflammasome in the kidneys of diabetic DsbA-L-deficient mice. **(A,B)** IHC staining reveals the increased expression of NLRP3 in the kidneys of diabetic DsbA-L^–/–^ mice. **(C,D)** Western blot analysis further confirms the increased expression of NLRP3 in the kidneys of diabetic DsbA-L^–/–^ mice. **(E–G)** The mRNA levels of IL-1β, IL-18, and caspase 1 in the different groups. **(H,I)** Western blot analysis reveals the expression of IL-1β and IL-18. The values are the mean ± SD. *n* = 3/group. *p < 0.05 compared with the control group; #*p* < 0.05 compared with the diabetic group.

Immunohistofluorescence staining revealed the notably upregulated expression of NLRP3 in the kidneys of DsbA-L^–/–^ or diabetic mice compared with the control although it was further increased in the DsbA-L^–/–^+HFD+STZ mice (shown in Figures 2A,B). Moreover, Western blot analysis showed a similar result, namely, that the kidneys of DsbA-L^–/–^+HFD+STZ mice had the highest expression level of NLRP3 compared with the other mice (shown in Figures 2C,D). In addition, the mRNA levels of IL-1β, IL-18, and caspase 1, signaling molecules downstream of the NLRP3 inflammasome, were also detected by PCR. Consistent with NLRP3 expression, the IL-1β, IL-18, and caspase 1 mRNA levels were notably increased in DsbA-L^–/–^ or diabetic mice, and their levels were further increased in DsbA-L^–/–^+HFD+STZ mice (shown in Figures 2E–G). Moreover, Western blot analysis showed a similar result, namely, that IL-1β and IL-18 expression was increased in DsbA-L^–/–^ or diabetic mice and further exacerbated in DsbA-L^–/–^ diabetic mice (shown in Figures 2H,I).

### Downregulated Expression of p-AMPK in the Kidney of Diabetic DsbA-L-Deficient Mice

**FIGURE 3:**
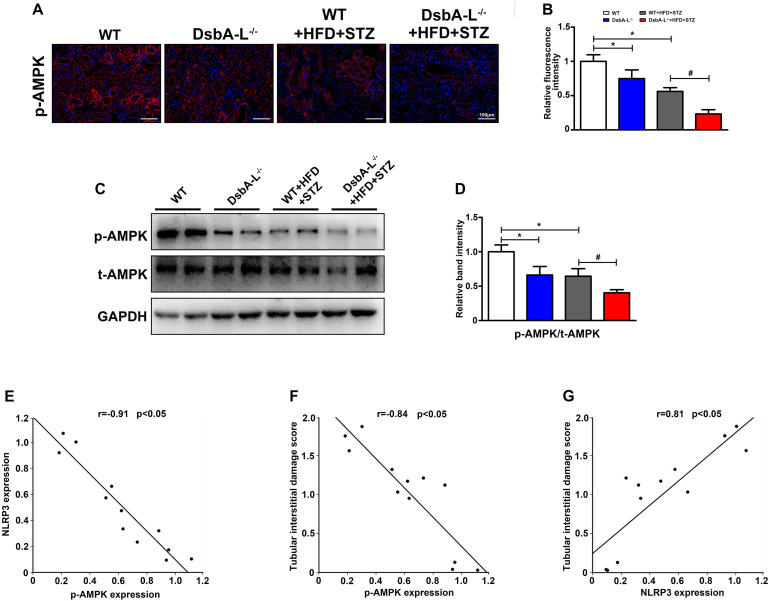
Inhibited AMPK phosphorylation in the kidneys of diabetic DsbA-L-deficient mice. **(A,B)** IHC staining reveals decreased expression of NLRP3 in the kidneys of diabetic and DsbA-L^–/–^ mice, which was further reduced in diabetic DsbA-L^–/–^ mice. **(C,D)** Western blot analysis further confirms the decreased AMPK phosphorylation in the kidneys of diabetic DsbA-L^–/–^ mice. Correlation analysis between AMPK phosphorylation and NLRP3, *r* = –0.91, *p* < 0.05 **(E)**, AMPK phosphorylation and tubular damage, *r* = –0.84, *p* < 0.05 **(F)**, and NLRP3 expression and tubular damage, *r* = 0.81, *p* < 0.05 **(G)**. The correlation of the two numerical variables was tested by Pearson’s analysis. The values are the mean ± SD. *n* = 3/group. *p < 0.05 compared with the control group; #*p* < 0.05 compared with the diabetic group.

With IHF staining, significantly downregulated expression of p-AMPK was observed in the kidneys of DsbA-L^–/–^ or diabetic mice compared with the kidneys of control mice, and it was further decreased in those of diabetic mice deficient in the DsbA-L^–/–^ gene (shown in Figures 3A,B). In addition, Western blotting analysis also revealed substantially decreased expression of p-AMPK in diabetic DsbA-L^–/–^ mice compared with other mice (shown in Figures 3C,D). Correlation analyses showed a negative correlation between the expression of p-AMPK and NLRP3 (*r* = −0.91, *p* < 0.05) (shown in Figure 3E) and tubular damage (*r* = −0.84, *p* < 0.05) (shown in Figure 3F). In addition, there was also a positive correlation between the expression of NLRP3 and tubular damage (*r* = 0.81, *p* < 0.05) (shown in Figure 3G).

### Effect of High Glucose (HG) Condition on p-AMPK and NLRP3 Expression in HK-2 Cells

**FIGURE 4:**
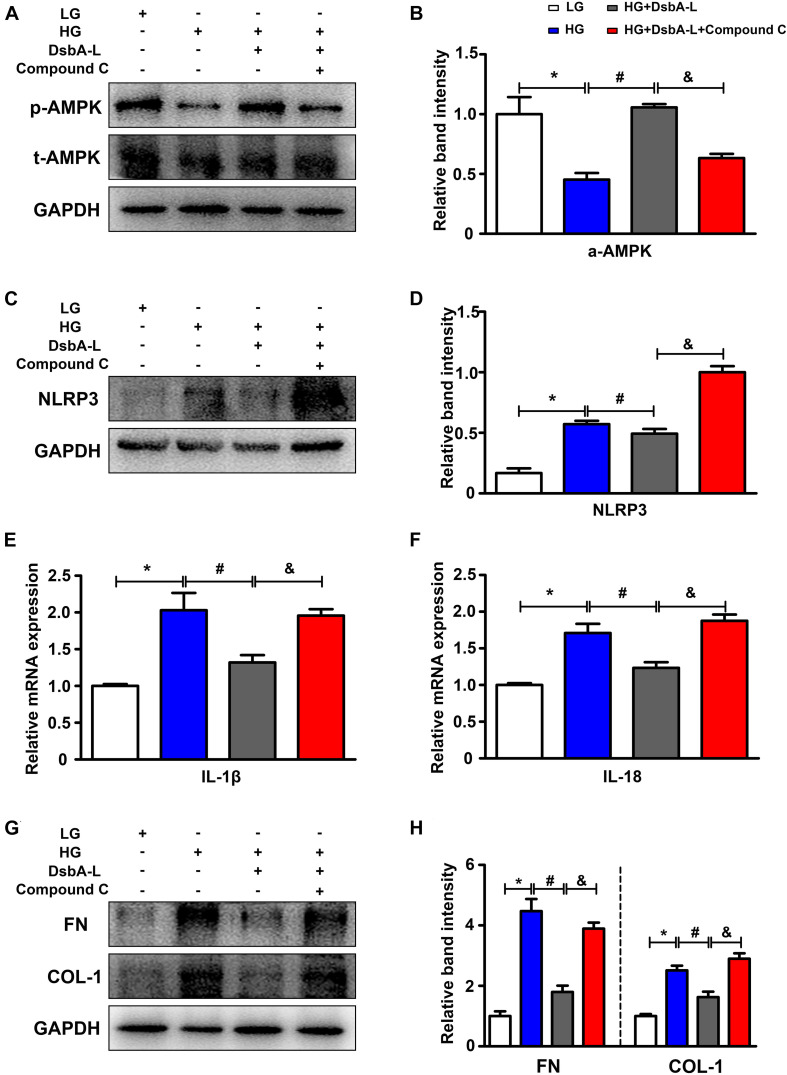
Altered AMPK phosphorylation and NLRP3 expression in HK-2 cells treated with HG treatment. **(A,B)** The expression of p-AMPK and total AMPK in HK-2 cells exposed to different treatments. **(C,D)** The expression of NLRP3 in HK-2 cells exposed to different treatments. **(E,F)** The mRNA level of IL-1β and IL-18 in HK-2 cells exposed to different treatments. **(G,H)** The expression of FN and COL-1 in HK-2 cells exposed to different treatments. The values are the mean ± SD. *n* = 3/group. *p < 0.05 compared with the LG group; #*p* < 0.05 compared with the HG group; &*p* < 0.05 compared with the HG +DsbA-L group.

DsbA-L expression was notably decreased in the high glucose (HG) group compared with the control (Supplementary Figure 1B). Western blot analysis was used to assess the effect of HG on p-AMPK expression. The expression of p-AMPK was significantly decreased in HK-2 cells treated with HG compared with the LG group (shown in Figure 4A, panel 1, lines 2 vs 1). There were no discernible changes in HK-2 cells treated with 30 nM mannitol, which served as the osmotic control (data not included). Overexpression of DsbA-L alleviated the reduced p-AMPK expression caused by HG (shown in Figure 4A, panel 1, lines 3 vs 2, Figure 4B). Furthermore, the DsbA-L-restored p-AMPK expression in response to HG was abolished by treatment with compound c (shown in Figure 4A, panel 1, lines 4 vs 3, Figure 4B). There was no significant difference in the expression of total AMPK and GAPDH (shown in Figure 4A, panels 2 and 3). In addition, the expression of NLRP3 (shown in Figures 4C,D), IL-1β, IL-18 (shown in Figures 4E,F), FN, and COL-1 (shown in Figures 4G,H) was increased by HG treatment compared with LG treatment. Interestingly, overexpression of DsbA-L restored the changes in NLRP3, IL-1β, IL-18, FN, and COL-1 expression induced by HG conditions (shown in Figures 4C–H). However, the protective effect of DsbA-L was inhibited by compound c treatment (shown in Figures 4C–H).

## Discussion and Conclusion

Activated renal inflammation and tubulointerstitial fibrosis were correlated with renal tubular cell injury, and this process involves the participation of multiple signaling pathways. NLRP3-mediated inflammatory activation is a significant event in DN and is believed to be involved in the occurrence and development of DN (Zhu et al., 2018). Unfortunately, the exact molecular mechanisms underlying these events are not yet understood and, therefore, require further study. In this study, we demonstrate that the disordered DsbA-L/p-AMPK/NLRP3 pathway is involved in the development of DN.

The NLRP3 inflammasome is a complex that consists of NLRP3, apoptosis-associated speck-like protein containing a CARD (ASC), and caspase 1 (Gurung and Kanneganti, 2015; Jo et al., 2016). Once the NLRP3 inflammasome is activated, it promotes the conversion of pro-IL-18 and pro-IL-1β to biologically active IL-18 and IL-1β, thereby promoting inflammation (Shao et al., 2015; He et al., 2016). As the most widely distributed inflammasome, NLRP3 is shown to be involved in the occurrence of neurological (Song et al., 2017) and cardiovascular diseases (Tong et al., 2020). Similarly, the activation of the NLRP3 inflammasome is also critical for the development of DN. El-Horany et al. (2017) demonstrate that the mRNA levels of NLRP3 and IL-1β are notably unregulated in diabetic patients with macroalbuminuria and are positively correlated with UACR. Similar results were observed in *in vivo* and *in vitro* studies. Overactivation of the NLRP3 inflammasome was observed in the kidneys of DN mice or HK-2 cells treated with HG (Han et al., 2018). By inhibiting the expression of NLRP3, HG-induced glomerular hypertrophy and sclerosis, mesangial dilatation, and interstitial fibrosis were alleviated in the kidneys of mice with DN. These findings strongly suggest that activation of the NLRP3 inflammasome accelerates renal damage in DN (Wu et al., 2018). In this study, we observed increased activation of the NLRP3 inflammasome in the kidneys of mice with STZ-induced diabetes compared with the control, which was accompanied by an increase in the mRNA levels of IL-1β, IL-18, and caspase 1. This phenomenon was further confirmed in HK-2 cells treated with HG. It is generally believed that increased blood glucose levels promote an increase in reactive oxygen species in tubular cells by causing mitochondrial dysfunction, which eventually leads to the activation of the NLRP3 inflammasome (Gao et al., 2019). Moreover, HG also activated the NLRP3 inflammasome by promoting the expression of NF-κB in the kidneys of mice with DN (Samra et al., 2016). However, these hypotheses cannot fully elucidate the mechanism by which NLRP3 is activated in the kidneys of DN.

DsbA-L is an antioxidant enzyme that exerts detoxification in lipid peroxides (Yang M. et al., 2019), and it can also maintain normal cellular homeostasis by inhibiting endoplasmic reticulum stress (Liu et al., 2015). DsbA-L plays a protective role in metabolic diseases, including DN (Gao et al., 2020). In previous studies, we demonstrate that DsbA-L alleviates renal cell apoptosis in DN by maintaining the integrity of MAMs (the coupling between mitochondria of the ER) (Yang M. et al., 2019). Moreover, we also confirm that DsbA-L improves lipid deposition and renal function through the AMPK pathway in DN (Chen et al., 2019). AMPK is an energy sensor that is present in all eukaryotic cells and is a heterotrimeric complex consisting of α, β, and γ subunits (Hardie et al., 2016). In addition to playing a central role in regulating energy metabolism, it is also involved in NLRP3 inflammasome activation. Hu et al. (2020) demonstrate that activated AMPK could alleviate AGE-induced endothelial NLRP3-mediated inflammation and oxidative stress. Moreover, suppressed p-AMPK levels and increased NLRP3 inflammasome activation were observed in the rat model of middle cerebral artery occlusion (MCAO), and intraperitoneal administration of Qingkailing (a traditional Chinese medicine) increased the phosphorylation of AMPK, thus suppressing the NLRP3 inflammasome activation (Ma et al., 2019). These findings suggest that phosphorylated AMPK inhibits activation of the NLRP3 inflammasome. In this study, we observed that phosphorylated AMPK was significantly decreased in the kidneys of diabetic DsbA-L-knockout mice or HK-2 cells treated with HG, which was accompanied by increased NLRP3 inflammasome activation, and decreased AMPK phosphorylation was associated with renal function. To further validate the DsbA-L/AMPK/NLRP3 signaling pathway, we overexpressed DsbA-L with a plasmid and noted that increased expression of DsbA-L restored the HG-induced decreases in AMPK phosphorylation and NLRP3 inflammasome activation. To verify whether DsbA-L inhibits activation of the NLRP3 inflammasome by p-AMPK, we treated cells with compound c, an inhibitor of AMPK phosphorylation (Dasgupta and Seibel, 2018; Chiou et al., 2020). Interestingly, we noted that compound c inhibited DsbA-L-mediated AMPK phosphorylation and exerted anti-inflammatory effects during treatment with HG. These results indicate that DsbA-L exerts its anti-inflammatory effect by promoting AMPK phosphorylation.

Although there are many questions that need to be answered, some key questions remain. For instance, what is the precise molecular mechanism by which DsbA-L regulates AMPK phosphorylation? Does overexpression of DsbA-L in animal models reduce NLRP3-mediated inflammation in the kidney? Here, we demonstrate that DsbA-L alleviates renal NLRP3-mediated inflammatory damage by promoting AMPK phosphorylation. We believe that this work explains the pathogenesis of DN from a new perspective.

## Data Availability Statement

The raw data supporting the conclusions of this article will be made available by the authors, without undue reservation.

## Ethics Statement

The animal study was reviewed and approved by the Medical Ethics Committee of Central South University.

## Author Contributions

MY designed the study, analyzed the data, interpreted the results, and drafted the manuscript. SL, NJ, XW, YH, HZ, XX, YL, CZ, and XZ contributed to the data collection and manuscript revision. LS was the corresponding author and was involved in the study design, data interpretation, and manuscript revision. All authors contributed to the article and approved the submitted version.

## Conflict of Interest

The authors declare that the research was conducted in the absence of any commercial or financial relationships that could be construed as a potential conflict of interest.

## Supplementary Material

Supplementary Figure 1 The expression of DsbA-L **(A)** *in vivo* and **(B)** *in vitro*.
