# Supplementary figures and images for: DsbA-L Ameliorates Renal Injury Through the AMPK/NLRP3 Inflammasome Signaling Pathway in Diabetic Nephropathy
Source: Front Physiol. 2021 Apr 30;12:659751. doi: 10.3389/fphys.2021.659751 (PMC8120163; doi:10.3389/fphys.2021.659751)

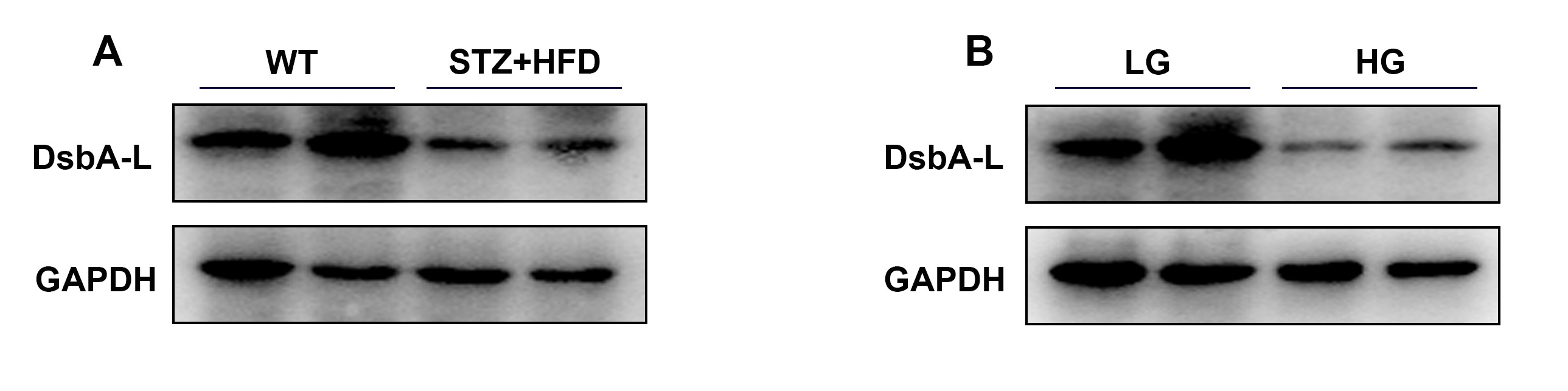

Supplement: Supplementary Figure 1 — The expression of DsbA-L (A) in vivo and (B) in vitro. [file Image_1.JPEG]
